# Learning fine-grained estimation of physiological states from coarse-grained labels by distribution restoration

**DOI:** 10.1038/s41598-020-79007-5

**Published:** 2020-12-15

**Authors:** Zengyi Qin, Jiansheng Chen, Zhenyu Jiang, Xumin Yu, Chunhua Hu, Yu Ma, Suhua Miao, Rongsong Zhou

**Affiliations:** 1 Department of Electronic Engineering, Tsinghua University, Beijing, 100084 China; 2 School of Aerospace Engineering, Tsinghua University, Beijing, 100084 China; 3 Tsinghua University Yuquan Hospital, Beijing, 100043 China

**Keywords:** Machine learning, Electrodiagnosis, Biomedical engineering

## Abstract

Due to its importance in clinical science, the estimation of physiological states (e.g., the severity of pathological tremor) has aroused growing interest in machine learning community. While the physiological state is a continuous variable, its continuity is lost when the physiological state is quantized into a few discrete classes during recording and labeling. The discreteness introduces misalignment between the true value and its label, meaning that these labels are unfortunately imprecise and coarse-grained. Most previous work did not consider the inaccuracy and directly utilized the coarse labels to train the machine learning algorithms, whose predictions are also coarse-grained. In this work, we propose to learn a precise, fine-grained estimation of physiological states using these coarse-grained ground truths. Established on mathematical rigorous proof, we utilize imprecise labels to restore the probabilistic distribution of precise labels in an approximate order-preserving fashion, then the deep neural network learns from this distribution and offers fine-grained estimation. We demonstrate the effectiveness of our approach in assessing the pathological tremor in Parkinson’s Disease and estimating the systolic blood pressure from bioelectrical signals.

## Introduction

Machine learning based algorithms^1–8^ have been recognized as promising enablers of computer-aided diagnosis and smart healthcare systems, since these algorithms are suitable for capturing the complex features and latent information in the recorded medical data to *estimate the physiological states* of the subjects. For instance, a recent work^9^ proposes a data efficient similarity learning algorithm that uses surface electromyography (sEMG) to classify the Parkinson’s Disease tremor into 5 levels with state-of-the-art accuracy. Researchers have also proposed effective time-frequency feature extraction and learning algorithms^10–12^ to estimate the neuromuscular disorders with EMG signals. Research^13–16^ has also been conducted to use novel convolutional neural networks to classify the sleeping stage of the subjects using the electroencephalogram (EEG) of their brains. EEG, EMG and sEMG are examples of medical data. Tremor severity, sleep stage and neuromuscular disorders are examples of physiological states that the machine learning models are employed to estimate. Normally, when we build such a machine learning model, we need a set of labeled data to train the model before it can perform the desired diagnostic task. Each input (e.g., a sequence of EMG) is labeled with the physiological state it implies (e.g., one of the stages of sleep). The labels are provided by human annotators, and the quality of these labels can have a significant influence on the model performance.

A typical problem faced by many of these machine learning algorithms is that the labels that they learn from are coarse-grained. In various scenarios the labels are provided as classification labels. By learning from such labels, the machine learning model takes the raw medical data as input and classifies the physiological state into discrete classes. Nevertheless, since the physiological state is inherently continuous, the discrete labels provided by human annotators may not be fine-grained enough to precisely reflect the physiological state. For instance, consider the simplest case where we classify the severity of a certain disease on a patient into two levels, *slight* and *severe*. In this sense, the annotators are required to provide binary labels by quantizing their judgments into two discrete categories. However, since the severity of a disease is actually a continuous variable, the binary quantization unavoidably introduces misalignment between the label and its true value. As a consequence, the model trained with such labels is not able to make precise predictions. Physiological states should be represented by real-valued continuous variables instead of discrete classes. One may argue that we can define more and finer classes to solve the problem, so that regression can be used instead of classification. However, obtaining precise ground truth is extremely difficult since in most cases, expert annotators label the physiological states based on their subjective clinical observation, rather than a ruler with precise scales.

**Figure 1:**
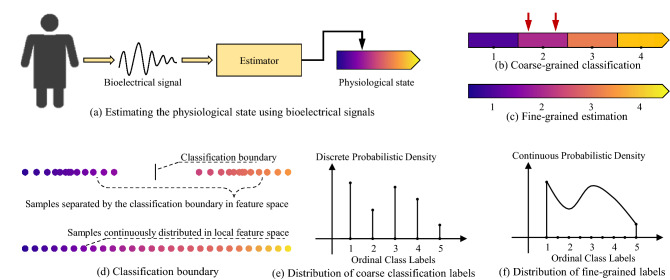
Overview. The basic objective is to estimate the intrinsic physiological state of subjects from their bioelectrical signals, illustrated in (**a**). (**b**) Shows an example of coarse labels, where the red arrows point to different states but have the same label. The inaccuracy is caused by quantizing the continuous physiological state (**c**) into discrete stages in (**b**). Although imprecise, the coarse labels are easy to obtain and abundant in quantity. The final objective is to offer a precise estimate of the real physiological state by learning from those coarse labels. (**d**) Compares the typical classification and the proposed fine-grained assessment. Classifiers tend to build boundaries in feature space, while there should be no absolute boundary between neighboring physiological states. (**e**) Shows an example distribution of discrete classification labels suffering from inaccuracy in describing continuous physiological states. (**f**) Presents the approximated distribution of continuous fine-grained labels, which is restored from (**e**) via interpolation by assuming the ordinal and continuous nature of physiological states.

In this work, we are interested in developing a general machine learning method to learn the fine-grained estimation of physiological state from coarse labels, which has notable benefits in exploring the rich details of physiological rhymes. Figure 1a illustrates the high-level framework, where we aim to build an estimator that takes the bioelectrical signal as input and predicts the fine-grained physiological states as *real-valued continuous variables*. The smooth color gradients indicate that the physiological states should change continuously. Figure 1b illustrates the classification labels that quantize the continuous physiological states (see Fig. 1c) into coarse-grained discrete classes. Developing machine learning algorithms that learn coarse labels to make fine-grained predictions is difficult, because the information the algorithm aims to learn is not explicitly provided by the labels in the training set. In previous literature, the research most relevant to ours is using Gaussian Process based approaches to learning fine-grained estimation from aggregate outputs^17,18^. They assume that the aggregate output or group statistics (e.g., the average fine-grained label) of a bag of inputs is known. Nevertheless, in our scenario, what we have is only the coarse label of each input, rather than the aggregation (e.g., the average) of their fine-grained labels. Therefore, these Gaussian Process based methods are targeted at a task intrinsically different from ours and cannot be used to directly solve our task.

Our approach is based on a mathematically rigorous theorem that we propose, which proves that such a task is achievable if the predictions of the machine learning algorithm satisfy two conditions. Based on this understanding, we propose the distribution restoration and ordinal-scale learning method to train the machine learning model so that the two conditions can be approximately satisfied. The proposed method is easy to implement using simple loss functions, yet effective in diverse tasks. More details are described in the “Method” section. Extensive experiments have been conducted on tremor severity estimation, parameter estimation on synthetic signals and systolic blood pressure estimation. The quantitative results demonstrate the superior performance of our method in learning fine-grained estimate of physiological states from coarse-grained labels.

### Remark 1

Physiological states as considered as continuous variables in this article. Typical examples include the severity of a disease, the blood pressure and the frequency of heartbeats. In some situations the states are discrete, but they still have their continuous measurements. For instance, heartbeats are discrete, but the frequency of heartbeats is continuous. By choosing a suitable definition of the physiological states, we can ensure that the states are viewed as continuous in these scenarios.

### Remark 2

This work mainly studies ordinal physiological states. While in some cases the states can bifurcate instead of being ordinal, our method is still applicable by incorporating with a classifier. For example, when the physiological state bifurcates into two branches corresponding to different types of diseases, we can first classify which branch the physiological state is on, then apply our method to that branch to perform fine-grained estimation of the disease severity.

## Results

We conduct comprehensive experiments on three specific tasks: (1) tremor severity assessment for Parkinson’s Disease (PD) using surface electromyography (sEMG), (2) parameter estimation for synthetic sEMG signals and (3) systolic blood pressure estimation using photoplethysmography (PPG) and electrocardiogram (ECG) signals. To the best of our knowledge, this work represents a pioneering attempt on the fine-grained assessment of bioelectrical states by learning from coarse labels.

### Tremor severity estimation

**Figure 2:**
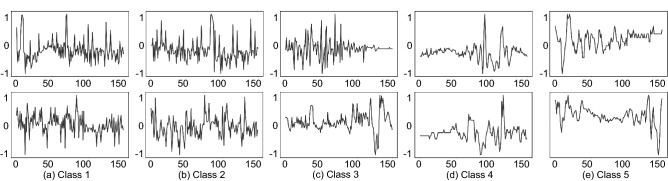
Visualization of PD-sEMG dataset. Each sEMG sequence is annotated with a class label among the five classes defined by the internationally accepted MDS-UPDRS^19^ rating scale. The class label indicates the tremor severity where Class 5 represents severe and Class 1 represents normal. It is clear that the sEMG features change gradually from Class 1 to Class 5 corresponding to the fact that the physiological states are naturally continuous. The discrete classification labels are only coarse approximations of the real situation and cannot capture the smooth transitions between neighbouring classes.

Tremor is a typical movement disorder occurring on the limbs of patients with Parkinson’s Disease. According to the universally accepted MDS-UPDRS^19^, the tremor severity is divided into 5 escalating levels, *normal, slight, mild, moderate, severe*, which we represent with integers $\{1, 2, 3, 4, 5\}$ respectively. The main evaluation is done on PD-sEMG^9^ dataset containing 10K sequences of single-channel sEMG collected from the upper limbs of 147 individuals at a sampling rate of 1 KHz. Figure 2 visualizes typical samples from the dataset. Each sample was annotated by multiple experts independently, and would not be used if the annotations were different, leading to generally unbiased labeling. In this experiment, the physiological state refers to the tremor severity.

#### Comparison to state-of-the-art methods

We cannot directly compare our approach with existing methods^10–13,20–23^, which focus on multi-class classification instead of fine-grained regression. For comparison, we round the predicted real-valued tremor severity to the nearest integers. We present the recall, precision for each class and the average accuracy in Table 1. Results are reported on the *test* set corresponding to^9^. FE and SL are short for feature engineering and similarity learning respectively. The proposed approach outperforms others under various evaluation metrics.

#### Fine-grained estimation of tremor severity

Our ultimate goal is not building a new multi-class classifier. Despite the classification performance shown in Table 1, we are not able to directly validate the correctness of the decimal part of our outputs because of the absence of fine-grained ground truth. However, it is still possible to estimate the *lower bound performance*, which occurs when the model is predicting an instance lying on the boundary between neighbouring classes. We implement this idea by assuming that the ground truth of class *i* is missing. We train the model using input signals of class $\{\ldots , i-1, i+1, \ldots \}$ and test the model using those of class *i*, which lies on the boundary of class $i-1$ and $i+1$ because of the ordinal nature of classes. The evaluation results are shown in Table 2. Results are reported on the *test* set corresponding to^9^. The precision is calculated by rounding the network output to the nearest integer to obtain the classification results, and then counting the classification precision for Class *i*. The pseudo MAE refers to the average of $|{\tilde{c}}_i - c^c_i|$, where ${\tilde{c}}_i$ is the prediction and $c^c_i$ is the coarse label that is regarded as pseudo ground truth. The model is trained using labels of class $\{\ldots , i-1, i+1, \ldots \}$ and evaluated on class *i*, the boundary between class $i-1$ and $i+1$. This experiment indirectly verifies the fine-grained output ${\tilde{c}}_i$. The slack L1 loss is always applied in training, and the two distribution losses both contribute to the performance according to the table. Even though the labels for class *i* are not provided, it is shown that the model still manages to predict the unseen instances of this class. Likewise, if we train the model using all the data of classes $\{1, 2, 3, 4, 5\}$, the decimal part of prediction ${\tilde{c}}$ is supposed to match the inaccessible fine-grained ground truth.

**Table 1:** Comparison to state-of-the-arts on PD-sEMG dataset.

Method	Recall (%)					Precision (%)				
	Class 1	Class 2	Class 3	Class 4	Class 5	Class 1	Class 2	Class 3	Class 4	Class 5
Bayes + FE^24^	72.25	47.50	55.25	22.00	52.50	46.69	63.33	56.09	29.73	53.71
MLP + SL^9^	67.00	68.50	26.75	73.50	99.50	62.04	56.61	84.92	81.00	66.90
S-Net + SL^9^	81.00	**93.25**	85.00	94.00	99.50	85.49	87.55	90.43	96.90	92.34
BioeNet (Ours)	**93.75**	90.50	**99.75**	**100.0**	**100.0**	**91.01**	**93.78**	**99.50**	**99.75**	**100.0**

The best performance in each column is marked in bold.

**Table 2:** Fine-grained estimation on PD-sEMG dataset.

Loss configuration		Precision (%)			Pseudo MAE		
${\mathscr {L}}_m$	${\mathscr {L}}_r$	$i = 2$	$i = 3$	$i = 4$	$i = 2$	$i = 3$	$i = 4$
$\checkmark$		75.58	58.33	49.64	0.937	1.015	0.838
$\checkmark$	$\checkmark$	**81.50**	**86.34**	**71.57**	**0.528**	**0.623**	**0.674**

The best performance in each column is marked in bold.

**Figure 3:**
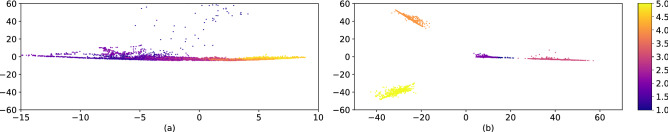
Interpretation in feature space. Each dot represents a sequence of bioelectrical signal in feature space obtained by executing PCA to the output of the forth convolutional layer from the end of BioeNet (see “Method” section for the detailed description of BioeNet). (**a**) Represents the case where the proposed training method is applied, while in (**b**) the network is trained as a typical classifier. The color represents the estimation of physiological state $c\in [1, 5]$. In (**a**), the distances among samples correspond well to their distance in real physiological states, while in (**b**) the continuity is broken, contradicting the fact that the physiological state should be continuous in nature.

**Figure 4:**
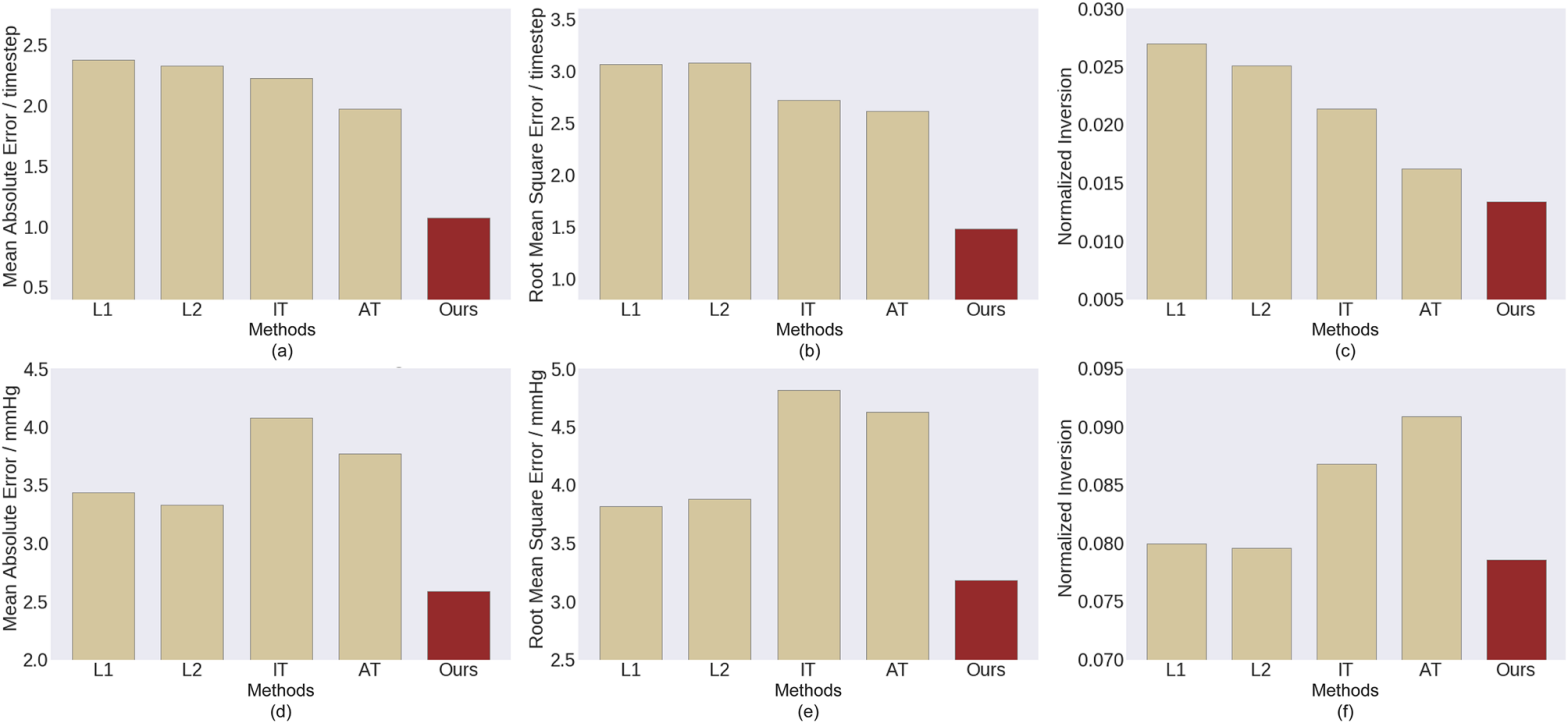
Estimation errors. The first row is for the parameter estimation of synthetic signals, and the second row is for the systolic blood pressure estimation.

**Figure 5:**
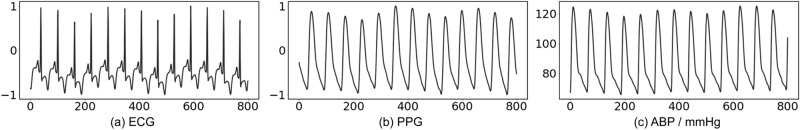
Visualization of the Cuff-less blood pressure estimation dataset. The Electrocardiography (ECG) and Photoplethysmography (PPG) are normalized for better visualization. The Systolic Blood Pressure (SBP) refers to the peak of Arterial Blood Pressure (ABP).

#### Feature space interpretation

In Fig. 1d, we have pointed out that typical classification model tends to build a boundary separating the features of different classes. This intuition is supported by Fig. 3b, where the samples are grouped around class centers, leaving a large gap among those centers. In this sense, because the intra-class separability is weakened, the model only learns a coarse estimation of physiological state. On the contrary, by employing the distribution loss, the distance in feature space is well correspondent with the distance in clinimetric scale as is shown in Fig. 3a. The boundaries are almost eliminated, corresponding to the fact that physiological states are generally continuous instead of being separated by sharp boundaries.

### Parameter estimation on synthetic signals

A disadvantage of the experiment of tremor severity estimation is that the fine-grained ground truth is unavailable, which means we can only indirectly examine the effectiveness of our approach. In this subsection we perform a new experiment using synthesized data whose fine-grained ground truth is accessible, which allows us to directly compare the network outputs with the ground truth. Based on the previous work^25^, we synthesized sEMG signals with controllable parameters, which are regarded as the fine-grained ground truth to be learned. The previous study^26^ has revealed that the wavelength of sEMG signals is correlated with the physiological states such as the frequency of pathological tremor. Therefore, we choose wavelength as the parameter to be estimated. Within a sequence of synthetic signal (i.e., a training or testing example), the wavelength is set to a constant floating point number generating the signal. 90K training sequences and 10K testing sequences are generated with wavelength uniformly distributed in $\left[ 150, 250\right]$. During training, we evenly divide the range into 5 intervals. The coarse label of sequences in an interval is set to the medium of the interval. In testing, the network is expected to predict the fine-grained label of each testing sequence.

#### Evaluation results

We consider four baseline methods including the most commonly used L1 regression (L1) and L2 regression (L2), as well as the immediate-threshold regression (IT)^27^ and all-threshold ordinal regression (AT)^27^. We evaluate the mean absolute error (MAE), root mean square error (RMSE) and the normalized inversion. Denote the fine-grained label of two sequences as $c_i$ and $c_j$, and the predicted label as ${\tilde{c}}_i$ and ${\tilde{c}}_j$. An inversion refers to the case where $c_i < c_j$ and ${\tilde{c}}_i > {\tilde{c}}_j$, or $c_i > c_j$ and ${\tilde{c}}_i < {\tilde{c}}_j$. The normalized inversion refers to the number of inversions divided by the maximum possible number of inversions that equals to $$\bigl ({\begin{matrix} n \\ 2 \end{matrix}}\bigr )$$, where *n* is the number of examples in the testing set. As is shown in the first row of Fig. 4, our method has the least fine-grained estimation error, exhibiting superior performance over the compared methods.

### Systolic blood pressure estimation

Here we perform an experiment on real data where the fine-grained ground truth is known, instead of using the synthetic data. The task is regressing blood pressure from bioelectrical signals. We adopt the cuff-less blood pressure estimation^28^ dataset where the Photoplethysmography (PPG), Electrocardiography (ECG) and corresponding Arterial Blood Pressure (ABP) are collected from at least 441 patients. Sampled data are visualized in Fig. 5. The network is to predict the maximum Systolic Blood Pressure (SBP) within a segment from the PPG and ECG signals. The fine-grained SBP ground truth is divided into 5 equally spaced coarse classes for training the deep neural network using the proposed framework. The baseline methods and evaluation criteria are the same as the experiment on synthetic signals.

#### Evaluation results

As is shown in the second row of Fig. 4, predictions made by the proposed method are the closest to the fine-grained ground truth and have the least estimation error. Our predictions also have the least normalized inversion. While a typical blood pressure value is between 80 and 120 mmHg, $92.97\%$ of our prediction errors are less than 5 mmHg, while using the all-threshold based ordinal regression approach this number decreases to $76.56\%$.

## Conclusions

In this article we propose a machine learning approach for predicting the fine-grained physiological states when only coarse-grained labels are given in the training data. Different from previous methods that aim to classify the physiological states into discrete classes, our method offers continuous and fine-grained estimation that are informative of even the slightest changes. Learning fine-grained predictions from coarse labels is intrinsically challenging due to the lack of supervision. Starting from mathematically rigorous proof, we reveal the possibility to solve this challenge by (1) restoring the continuous probability distribution of the fine-grained labels and (2) preserving the order of the fine-grained predictions. Then we propose a set of simple yet effective loss functions that enable the network outputs to approximately satisfy both conditions.

The fine-grained estimation of physiological states is potentially useful in a wide range of applications such as monitoring the physical condition of patients. Take the Parkinson’s Disease tremor as an example. The tremor is divided into 5 discrete levels by the MDS-UPDRS^19^. But this does not provide sufficient resolution to monitor the tremor severity in a fine-grained scale. After taking a medicine, the tremor might become less severe, but the difference could be too small to change the severity from one level to another. Our method reveals the possibility to automatically monitor the slight changes and provide more information, for example, on the effectiveness of medication.

To evaluate the proposed method, we conduct comprehensive experiments on tremor severity estimation using sEMG signals, systolic blood pressure estimation using PPG and ECG signals, as well as the parameter estimation from synthetic sEMG signals. Results have shown that the proposed approach can significantly reduce the regression errors. The effectiveness of each loss function we propose is also examined. Our algorithm has demonstrated potentials in automatically and precisely diagnosing diseases and monitoring the physical conditions of individuals in a more sophisticated way.

## Method

### Problem formulation

Given a sequence of stochastic bioelectrical signal $V=[{\mathbf {v}}_1, {\mathbf {v}}_2, \ldots , {\mathbf {v}}_L]$ with *L* sampling time steps, our objective is to learn a mapping function $f: V \rightarrow c$, where $c \in [1, C]$ is the assessment of the physiological pattern that *V* reflects. For example, *c* can represent the severity of tremor and *C* equals to the maximum severity level. In a typical classification setting, *c* is an integer belonging to the set $\{1, 2, \ldots , C\}$, as such the classification model is not designed to offer sufficient resolution to account for the intra-class variations, indicating that such classification is coarse-grained. Previous work discretized *c* into countable classes (e.g., 1, 2, $\ldots$, 5) and modeled $f: V \rightarrow c$ as a classification function. This formulation cannot give a fine-grained estimate of physiological states. For instance, when two patients are both of severity level 2, their actual severity can be slightly different (e.g., 2.1 vs 2.3) but the classification method cannot distinguish them. Instead, our aim is to provide a fine-grained estimate of the physiological state that can differentiate the slight differences. This is potentially useful in various scenarios. Suppose that a patient of severity level 2 took a medicine and then the severity decreased to 1 after 10 h. Using our fine-grained estimation, we can measure the small changes of the severity overtime, which could help physicians to understand the effect of the medicine. On account of the importance of fine-grained estimation, we first replace the discrete classification with the continuous regression so that *c* is a floating point number, which is expected to reasonably correspond with the naturally continuous physiological states. Nevertheless, it causes difficulties in training for the lack of ground truth labels. After all, it is over demanding for doctors, the annotators, to score their observation in accurate floating point numbers. The available ground truth data are only discrete integers indicating the categories. Hence we consider the problem as learning an estimator function *f* that maps *V* to a continuous real-valued *c* using typical classification labels, which we refer to as coarse-grained labels.

### Approximating the estimator function

**Figure 6:**
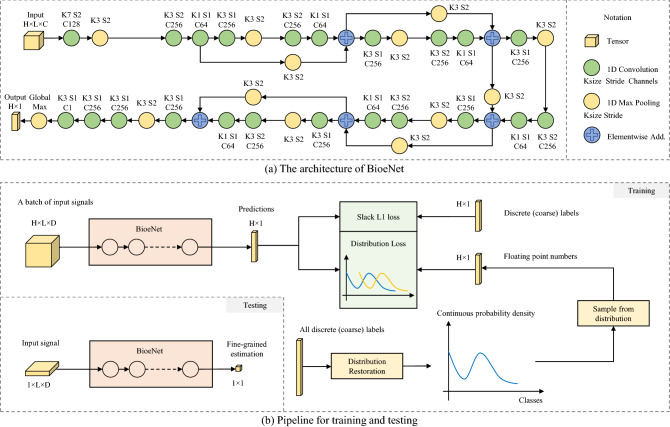
Network and pipeline overview. (**a**) illustrates the proposed BioeNet. Taking the bioelectrical signals as input, BioeNet predicts the physiological states indicated by these signals. The specific type of physiological states depends on training labels, for example, tremor severity. (**b**) Provides a systematic view of the training and testing process.

We propose a convolutional neural network BioeNet (see Fig. 6a for details) to approximate the function $f: V \rightarrow c$. BioeNet takes as input a batch of bioelectrical signals with length *L* and the number of channels *D*. It outputs a floating point number for each sequence of signal in a batch representing the estimated physiological state. The batch size is denoted as *H*. By using global max-pooling in the head layer, BioeNet is translation invariant to input signals. In the followings, we will show how the neural network can approximate the function *f* by learning from coarse labels. We will first propose a theorem that mathematical proves the feasibility of learning fine-grained estimation from coarse labels. Then we will propose two learning strategies, distribution restoration and ordinal scale learning, to implement the theory.

#### Theorem 1

*Two continuous probability density functions* $p_A(x)$ *and* $p_B(x)$ *are defined on* $x \in \left[ \theta _1, \theta _2 \right]$. $p_A(x) \ge \delta > 0$ *and* $p_B(x) \ge \delta > 0$. $S_A=\{a_1, a_2, \ldots , a_n \}$ *and* $S_B=\{b_1, b_2, \ldots , b_n\}$ *are independent random samples from* $p_A(x)$ *and* $p_B(x)$ *respectively. If satisfying (I)* $p_A(x) \equiv p_B(x)$ *and* $(II)~\forall i, j \in \{1, 2,\ldots , n\}$, $a_i\le a_j \Leftrightarrow b_i \le b_j$, *then* $\forall i\in \{1, 2,\ldots , n\}$ *we have*:

1 $$\begin{aligned} {\mathbf {E}}||a_i-b_i||^2 \le \frac{1}{2\delta ^2(n+2)} \end{aligned}$$

Here we propose Theorem 1 as the mathematical basis of our method and prove it at the end of this section. Note that Theorem 1 does not rely on a specific network architecture (e.g., BioeNet). Based on Theorem 1 we conclude that when *n* is sufficiently large, we have

2 $$\begin{aligned} \lim _{n\rightarrow \infty } {\mathbf {E}}~||a_i-b_i||^2 = 0, \end{aligned}$$

which means $S_B$ almost equals to $S_A$, or their element-wise error has zero expectation, if both conditions stated in Theorem 1 are satisfied. In our specific task, we consider $S_B$ as the network prediction and $S_A$ the fine-grained ground truth as floating point numbers. $S_A$ is inaccessible in training but is assumed to exist in reality. Our objective is to enable the network prediction $S_B$ to approximately comply with both of the conditions so that it is close to $S_A$.

### Distribution restoration

Meeting the first condition of Theorem 1 requires the probabilistic distribution of network predictions accord with that of the intrinsic physiological state *c*. As a continuous variable, *c* obeys a continuous distribution that can be restored from the discrete distribution of training labels as is in Fig. 1e by interpolation. Mathematically, there are a infinite number of curves that can interpolate the discrete points. In order to obtain a smooth curve and reduce redundancy, we utilize cubic interpolation here. The justification of interpolating neighboring stages comes from the rationality of clinical practices. Clinical experts define these stages to thoroughly describe a physiological process. If the experts had observed that a phenomenon seriously undermined the smoothness of the stage change, they should have already defined a new stage based on this phenomenon. Therefore, the changes between neighboring stages should be generally gradual and smooth. The physiological states are continuous because of the natural continuity of most physiological processes. Figure 1f shows the continuous probabilistic density obtained from the discrete distribution of coarse classification labels in Fig. 1e. The essence of this distribution restoration is making use of the *ordinal* information of class labels. In physiological state classification, neighboring classes can be assumed to be in a natural order. It should be noticed that this may not be true in other classification tasks such as object recognition in computer vision and emotional semantic analysis in natural language processing. Let $p_C(x)$ denote the restored distribution. Although $p_C(x)$ is only an approximation, it still provides richer information than the discrete classification labels. During training, we sample a batch of floating point numbers to represent $p_C(x)$ statistically, then calculate its Maximum Mean Discrepancy loss ${\mathscr {L}}_m$ with the network outputs

3 $$\begin{aligned} {\mathscr {L}}_m = \frac{1}{H^2}\sum _{i, j}^H \left[ k({\tilde{c}}_i, {\tilde{c}}_j) - 2k({\tilde{c}}_i, c^s_j) + k(c^s_i, c^s_j)\right] , \end{aligned}$$

where *H* is the batch size, ${\tilde{c}}_i$ (${\tilde{c}}_j$) denotes the network output and $c^s_i$ ($c^s_j$) indicates the floating point numbers sampled from the distribution $p_C(x)$; and *k*(*x*, *y*) represents the Gaussian kernel. This loss item minimizes the discrepancy between the distribution of network predictions and the continuous distribution restored from coarse labels. In order to avoid the network outputs to concentrate in the vicinity of integers, We also minimize the kurtosis of the outputs. The kurtosis loss ${\mathscr {L}}_r$ is formulated in Eq. (4), where $\mu _{{\tilde{c}}}$ and $\sigma _{{\tilde{c}}}$ indicate the forth-order expectation and variance of ${\tilde{c}}_i$ for class ${\hat{c}}$. The kurtosis of all classes are summed up to be optimized.

4 $$\begin{aligned} {\mathscr {L}}_r = \sum _{{\hat{c}}=1}^C \frac{\mu _{{\hat{c}}}}{\sigma ^2_{{\hat{c}}}}, \quad \mu _{{\hat{c}}} = {\mathbf {E}}\left[ ({\tilde{c}}_i - {\mathbf {E}}[{\tilde{c}}_i])^4|c^c_i={{\hat{c}}}\right] , \quad \sigma _{{\hat{c}}} = {\mathbf {E}}\left[ ({\tilde{c}}_i - {\mathbf {E}}[{\tilde{c}}_i])^2|c^c_i={{\hat{c}}}\right]. \end{aligned}$$

The *distribution loss* is the sum of ${\mathscr {L}}_m$ and ${\mathscr {L}}_r$. It functions as an implicit supervision. Although the ground truth value corresponding to ${\tilde{c}}_i$ cannot be directly obtained, we expect ${\tilde{c}}_i$s to comply well with the restored continuous probabilistic distribution $p_C(x)$.

### Ordinal scale learning

The second condition of Theorem 1 requires the element order in $S_B$ to be the same as that in $S_A$. A specific example in Fig. 1b involves two arrows indicating different physiological states. The network is expected to give the left one a lower score than the right one, preserving their real order. When there are sufficient many arrows, the second condition can be approximately satisfied. In training labels, though the instances of the same class are randomly permuted, the order of instances from different classes is provided by their coarse-grained labels. Hence we propose the following *Slack L1 loss* for the network to learn such an ordinal scale.

5 $$\begin{aligned} {\mathscr {L}}_l = \frac{1}{H}\sum _{i}^H \sqrt{max(|{\tilde{c}}_i - c^c_i|- \alpha , 0)^2 + \beta ^2} - \beta , \end{aligned}$$

where $c^c_i$ indicates the coarse classification label as integer; $\alpha$ is the tolerance range and $\beta$ is utilized to smooth the gradients. When the difference $|{\tilde{c}} - c^c|$ is less than a tolerance threshold $\alpha$, ${\mathscr {L}}_l$ equals to zero so that the network does not get punished. The basic concept underlying Eq. (5) is that, the coarse-grained label $c^c$ only represents the integer part of the real ground truth, and thus the difference $|{\tilde{c}} - c^c|$ may not equals to zero when ${\tilde{c}}$ is a floating point number. Therefore, we slack the objective to a tolerance range $\alpha$ to introduce more flexibility to the network, which learns a generalized ordinal regression without suffering from the discontinuity of training labels. Note that the order of instances within the same class is inaccessible due to the absence fine-grained ground truth. Even so, the network still manages to distinguish the intra-class order, which will be demonstrated in the experiment section.

### Pipeline overview

A systematic view of the learning pipeline is shown in Fig. 6b. During training, a mini-batch contains *H* sequences of bioelectrical signals, each with *L* timesteps and *D* channels. BioeNet predicts a score indicating the physiological state for each sequence of signal, thus yielding a $H\times 1$ vector, whose Slack L1 loss with the coarse-grained labels is computed. Meanwhile, we sample *H* numbers from the restored continuous distribution and compute their maximum mean discrepancy loss with the network outputs. The parameters of the convolution kernels in BioeNet are optimized via gradient descent. During testing (inference), BioeNet takes a sequence of bioelectrical signal and directly predicts the physiological state in an end-to-end fashion.

### Implementation details

The proposed BioeNet is implemented using Tensorflow and Python. All the convolutional layers are followed by a batch normalization layer and ReLU non-linearity, except that the last layer is purely linear. We choose $H=512, L=2048$ in training, while the number of channels *D* depends on the acquisition instruments of different bioelectrical signal datasets. We utilize Adam optimizer to train for 60 epochs at a learning rate $10^{-4}$ and then for 20 epochs at $10^{-5}$.

*Proof of Theorem 1* The proof consists of two parts. For the first part, let $y \sim \mathrm U(0, 1)$ and $z \sim \mathrm U(0, 1)$ be independent stochastic variables. $\{y_1, y_2, \ldots , y_n \}$ and $\{z_1, z_2, \ldots , z_n\}$ are two sets of independent samples of *y* and *z* with elements sorted in an ascending order, i.e., $\forall i \le j$, $y_i\le y_j$ and $z_i\le z_j$. It is well-known that $y_k$ and $z_k$ are the *k* th order statistic^29^ of the standard uniform distribution and the variance.

6 $$\begin{aligned} Var[y_k]=Var[z_k]=\frac{k(n-k+1)}{(n+1)^2 (n+2)}. \end{aligned}$$

Since $y_k$ and $z_k$ are independent, we have

7 $$\begin{aligned} {\mathbf {E}}~||y_k-z_k||^2&= {\mathbf {E}}~[y_k^2] + {\mathbf {E}}~[z_k^2] - 2~{\mathbf {E}}~[y_k]~{\mathbf {E}}~[z_k] \nonumber \\&= 2~{\mathbf {E}}~[y_k^2] - 2~{\mathbf {E}}~[y_k]^2 = 2~Var[y_k] \nonumber \\&\le \frac{(n+1)^2}{2(n+1)^2 (n+2)} = \frac{1}{2(n+2)} \end{aligned}$$

For the second part, we consider two continuous probability density functions $p_A(x)$ and $p_B(x)$ defined on $x \in \left[ \theta _1, \theta _2 \right]$. $p_A(x) \ge \delta > 0$ and $p_B(x) \ge \delta > 0$. $S_A=\{a_1, a_2, \ldots , a_n \}$ and $S_B=\{b_1, b_2, \ldots , b_n\}$ are independent random samples from $p_A(x)$ and $p_B(x)$ respectively, satisfying (I) $p_A(x) \equiv p_B(x)$ and (II) $\forall i, j \in \{1, 2,\ldots , n\}$, $a_i\le a_j \Leftrightarrow b_i \le b_j$, then $\forall i\in \{1, 2,\ldots , n\}$. Let ${\hat{a}}_k$ and ${\hat{b}}_k$ be the *k* *th* smallest elements in $S_A$ and $S_B$ respectively, and *F*(*x*) be the cumulative distribution of $p_A(x)$ that is the same as $p_B(x)$. Established on the fact that any continuous distribution can be mapped to the standard uniform by its cumulative distribution function, we can conclude that $S^F_A=\{F(a_1), F(a_2), \ldots , F(a_n) \}$ and $S^F_B=\{F(b_1), F(b_2), \ldots , F(b_n)\}$ both follow $\mathrm U(0, 1)$. Then we have

8 $$\begin{aligned} {\mathbf {E}}~||F({\hat{a}}_k)-F({\hat{b}}_k)||^2 \le \frac{1}{2(n+2)}, \end{aligned}$$

which is based on Eq. (7). And thus:

9 $$\begin{aligned} {\mathbf {E}}~||{\hat{a}}_k-{\hat{b}}_k||^2&= {\mathbf {E}}~||F^{-1}[F({\hat{a}}_k)]-F^{-1}[F({\hat{b}}_k)]||^2 \nonumber \\&\le {\mathbf {E}}~\left[ {\mathrm{Sup}}\left( \frac{dF^{-1}}{dx}\right) ^2~||F({\hat{a}}_k)-F({\hat{b}}_k)||^2\right] \nonumber \\&= \frac{1}{\delta ^2}~{\mathbf {E}}~||F({\hat{a}}_k)-F({\hat{b}}_k)||^2 \nonumber \\&\le \frac{1}{2\delta ^2(n+2)}. \end{aligned}$$

Since $\forall i, j \in \{1, 2,\ldots , n\}, a_i\le a_j \Leftrightarrow b_i \le b_j$, the ranking of $a_i$ in $S_A$ equals to that of $b_i$ in $S_B$. There exists a *k* that $a_i$ and $b_i$ are the *k* *th* smallest element in $S_A$ and $S_B$ respectively. The theorem is finally proved.

10 $$\begin{aligned} {\mathbf {E}}~||a_i-b_i||^2&= {\mathbf {E}}~||{\hat{a}}_k-{\hat{b}}_k||^2 \le \frac{1}{2\delta ^2(n+2)} \end{aligned}$$

## Data Availability

The PD-sEMG^9^ dataset, the synthetic sEMG dataset and the source code are available at https://github.com/Zengyi-Qin/fine-biostate. The Cuff-Less Blood Pressure Estimation^28^ dataset is publicly available online https://www.kaggle.com/mkachuee/BloodPressureDataset.
